# Physical Activity Level and Dietary Intake Associated with Fat-Free Muscle Mass Changes During Intentional Weight Loss in Overweight and Obese Subjects

**DOI:** 10.3390/nu16234044

**Published:** 2024-11-26

**Authors:** Salah Eldin Elnagi Gariballa, Ghada Al-Bluwi, Javed Yasin

**Affiliations:** Internal Medicine, College of Medicine & Health Sciences, United Arab Emirates University, Al Ain P.O. Box 17666, United Arab Emirates; ghadabluwi@uaeu.ac.ae (G.A.-B.); javed.yasin@uaeu.ac.ae (J.Y.)

**Keywords:** protein, calories, fruits and vegetables, body composition, inflammation, oxidative damage

## Abstract

Background: The prevalence of obesity and related complications is increasing relentlessly worldwide. The effect of intentional weight loss strategies for obese individuals on fat-free muscle mass (FFMM) and metabolic and general health is not well known. The aim of this research is to measure the effects of dietary intake and physical activity level on FFMM change during intentional weight loss in obese subjects. Materials and Methods: Nine hundred and sixty-five overweight and obese community free-living subjects had the effects of physical activity level and dietary intake on FFMM change during intentional weight loss assessed in a prospective longitudinal study. Anthropometric, physical activity, dietary intake, inflammatory markers, and oxidative damage were assessed at baseline and follow-up. Validated questionnaires were used to measure dietary intake and physical activity. We compared FFMM loss or gain between subjects stratified by calorie, protein, and fruit and vegetable intake and physical activity levels. The Cox proportional hazards analysis was used to determine the independent effects of dietary intake and physical activity on FFMM changes. Results: A total of 965 subjects [(mean (SD) age 39 ± 12 years, 801 (83%)] females] were assessed at baseline with follow-up for a period of 427 ± 223 days. Using the WHO criteria for body mass index (BMI), 284 (30%) subjects were found to be overweight and 584 (62%) were obese. We found significant correlations between fat–muscle mass ratio (FMR) and inflammatory and oxidative damage markers. After adjusting for important prognostic indicators, age, gender, occupation, physical activity, and fruit and vegetable consumption were found to be significantly associated with FFMM at baseline (*p* < 0.05). We found no statistically significant difference in dietary protein or amino acids intake in subjects who gained FFMM compared to those who lost FFMM both at baseline and follow-up. By contrast, high consumption of fruits and vegetable and increased calorie intake were associated with increased odds of FFMM gain (*p* < 0.05). Increased physical activity was independently associated with significant FFMM gain after adjusting for other important indicators ([hazard ratio (95% CI): 0.49 (0.25, 0.97); *p* = 0.039]. Conclusions: Increased physical activity and high calorie, fruit and vegetable intake are associated with FFMM preservation or gains during intentional weight loss in obese subjects.

## 1. Background

The rate of obesity is increasing relentlessly worldwide and poses a major risk factor for cardiovascular disease (CVD) including type 2 diabetes and hypertension [1,2,3]. For instance, in some developing countries and the Gulf region, obesity prevalence is increasing rapidly and is now a major public health problem [1,2,3,4]. Body mass index (BMI) is used to define overweight and obesity in adults because it correlates with associated morbidities. However, it has major limitations because it does not discriminate between body composition components including fat and muscle mass [5].

Studies have revealed associations between different elements of body composition and obesity-related health risks [6,7,8]. Generally, obese people have more fat and FFMM than normal weight individuals [9,10,11]. Furthermore, body composition varies among individuals with the same BMI, who often reveal diverse metabolic and cardiovascular risks [12]. Some of the reasons for these metabolic health discrepancies include differences in body composition, namely the amount of body fat in relation to FFMM [12]. Increased FFMM is thought to be protective and have positive health effects on risk factors associated with obesity. By comparison, increased fat mass is associated with increased adverse effects [6,7,8].

Weight loss is a key therapy for obesity because it mitigates or resolves metabolic risk factors associated with obesity. For example, it can mitigate important CVS risk factors such as high blood pressure, poor glycemic control, and an increased lipid profile [1,2,3]. To preserve FFMM, regular physical activity and high protein intake are recommended for intentional weight loss in obese people [9]. However, the potential benefits of weight loss as a result of dietary treatments are thought to be reduced by the associated weight loss-induced loss of FFMM, which can cause impaired muscle function, particularly in vulnerable populations such as older people. Furthermore, some recent evidence points to the opposite effect of a low-calorie diet and resistance exercise on FFMM [9]. Therefore, optimal weight loss should aim to increase or at least preserve FFMM size.

Overall, the effects of weight loss strategies in obese individuals on body composition constituents including fat mass in relation to FFMM and metabolic and general health are not well studied and, therefore, not well known [13].

In this longitudinal cohort study, we assessed the effects of dietary calories, protein, and fruit and vegetable intake and physical activity on FFMM size during intentional weight loss in overweight and obese individuals.

## 2. Material and Methods

Obese and overweight individuals referred to healthcare facilities for weight management were approached and asked to participate in the study. Following informed written consent and recruitment to the study, eligible patients had anthropometric measurements and a fasting blood sample taken for measurements of biological outcome markers. Participants received generic dietary education on weight loss/management strategies from a dietician or in a general medical clinic. The inclusion criteria include overweight and obese individuals referred for weight management. The exclusion criteria include individuals participating in other intervention trials or those with active clinical or psychiatric disease. The study was approved by the local research ethical committee. Subjects were otherwise managed according to standard practice. Participants were given the option of visiting the clinic for further advice on how to reduce or manage their weight.

### 2.1. Measurements

For recruitment, all participants had baseline data collection, including demographic and medical data, smoking, alcohol and drug intake, diet, physical activity, and anthropometric and biological measurements.

### 2.2. Anthropometric Body Composition Analysis

Height was measured without shoes in centimeters. Body weight was measured in light clothes in kilograms using a multifrequency Tanita. Body composition parameters including BMI, fat mass, and FFMM were measured in bare feet and light clothes using a standard Tanita M10T6360 analyzer (serial number 10050089, Tanita Corporation, Itabashi-ku, Tokyo, Japan). Participants were instructed to keep their bare feet on the marked area on the analyzer and firmly in contact with the analyzer surface. Results were printed onto a sheet. A validated Tanita bioelectrical impedance method was used to estimate fat and FFMM throughout the body [14,15].

Waist and hip circumferences were measured to the nearest 0.1 cm with a flexible plastic tape using standard methods.

Fruit and vegetable intake: A validated self-administered food frequency questionnaire was used to assess subjects’ fruit and vegetable intake. The questionnaire was developed and validated against a 7-day weighted dietary intake [16].

Calorie intake: Calorie intake was measured in a subgroup using 24 h recalls twice at baseline and follow up visits using a locally validated food frequency questionnaire and analyzed using a food composition database (http://www.tinuvielsoftware.com/).

Physical Activity: A validated questionnaire used in our previous studies was also used to assess physical activity both at home and at work. Data were obtained from the daily number of hours subjects spent doing housework [17].

Blood samples: Fasting blood samples were collected into tubes and centrifuged immediately for 10 min. Serum and plasma samples were stored at −80 °C for future analysis. Thiobarbituric Acid Reactive Substances (TBARS), a lipid peroxidation product, was measured using assay kit number 10009055 (1180 E. Ellsworth Rd., Ann Arbor, MI 48108, USA). Calorimetric assay kits numbers 703102 and 706002 were used to measure antioxidant glutathione (GSH) and TNF inflammatory markers. Protein carbonyl was determined using a reagent kit (10005020). Renal and liver functions, lipids, and C-reactive protein (hs-CRP) were measured using standard methods.

### 2.3. Statistical Power of the Study

Our sample, which followed 343 overweight and obese individuals with complete FFMM data, allowed the detection of 10% change in FFMM with 80% power to detect significant changes in FFMM of this magnitude at a *p*-value of less than 0.05.

### 2.4. Statistics Analysis

All data were analyzed using IBM SPSS version 29. Analysis-of-variance and the independent *t*-test were used to test within and between-subject differences, and a *p* value of ≤0.05 was considered significant. Logistic regression and the Cox proportional hazard models were used to examine the influence of age, gender, occupation, fruit and vegetable intake, and physical activity levels on the probability of FFMM gain or loss at baseline and follow-up, respectively. FFMM was dichotomized (i.e., loss vs. gain) and analyzed as the dependent variable against predictor variables. Odds of FFMM loss or gain were presented graphically using the Kaplan–Meier hazard curve.

## 3. Results

### 3.1. Baseline Characteristics

Nine hundred and sixty-five overweight and obese subjects [mean (SD) age 39 ± 12 years, 801 (83%) females] were included in the analysis. A total of 284 (30%) subjects were overweight compared to 584 (62%) obese individuals. Table 1 shows baseline characteristics stratified by BMI based on WHO cutoff diagnostic criteria for obesity. As expected, there are significant differences between obese and overweight subjects compared with normal weight subjects in age, gender, anthropometric measures including FFMM both at baseline and follow-up.

Table 2 shows significant differences in age, hip circumference, fat, and FFMM at baseline between overweight and obese male and female individuals.

### 3.2. Associations Between FFMM, Clinical, and Metabolic Prognostic Indicators

Table 3 shows significant correlations between baseline fat–muscle mass ratio (FMR) and metabolic risk factors including inflammatory and oxidative damage markers after adjusting for sex (*p* < 0.05). There were also significant associations between FMR and lipid profile variables; however, some of these subjects were already taking lipid-lowering medications.

The multiple logistic regression analysis revealed that age, gender, occupation, physical activity and fruit and vegetable consumption were independently associated with FFMM at baseline (*p* < 0.05), (Table 4).

### 3.3. Associations Between Dietary Intake and FFMM Gain/Loss at Follow-Up

**Figure 1:**
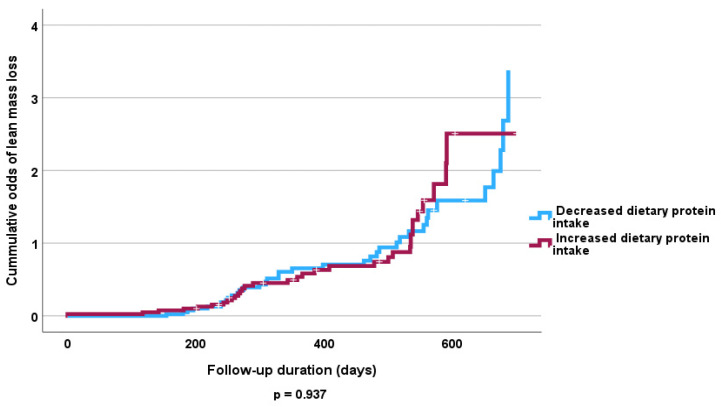
Kaplan–Meier curve of the effect of dietary protein intake at follow-up on FFMM loss.

**Figure 2:**
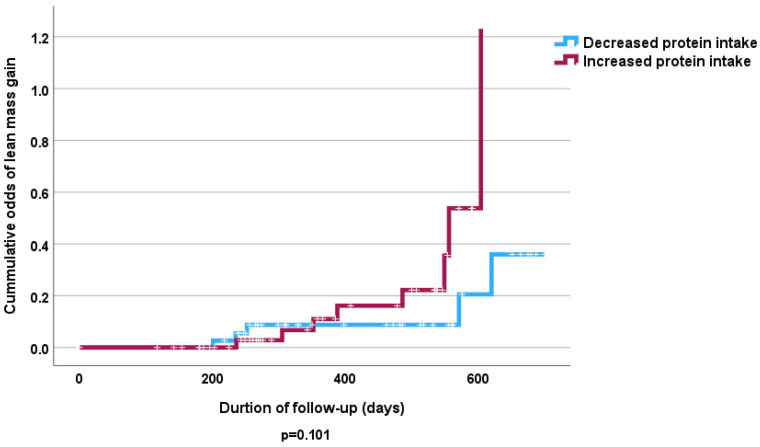
Kaplan–Meier curve of the effect of dietary protein intake at follow-up on FFMM gain.

We found no statistically significant difference in dietary protein and amino acid intake in subjects who gained FFMM compared with those who lost FFMM both at baseline and follow-up (Figure 1 and Figure 2). Baseline dietary protein intake was 50 (31) gm/day among those who lost FFMM compared to 43 (38) gm/day among those gained FFMM (*p* = 0.417). The corresponding protein intake at follow-up was 41 (27) gm/day and 41 gm (21) gm/day, respectively (*p* = 0.432).

**Figure 3:**
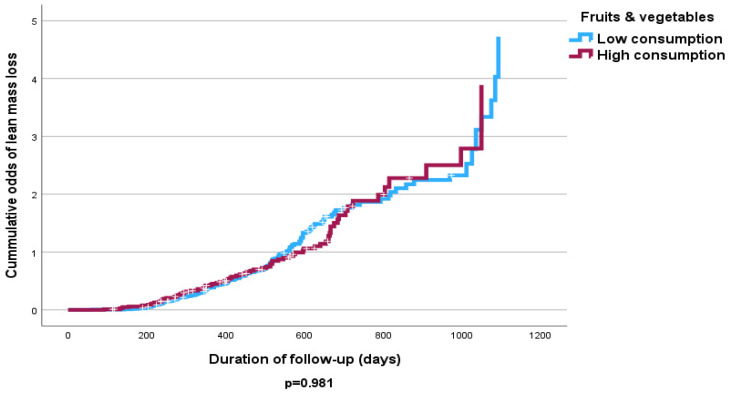
Kaplan–Meier curve of the effect of fruit and vegetable consumption on FFMM loss (high consumption >3.6 servings/day vs. low consumption <3.6 servings/day).

**Figure 4:**
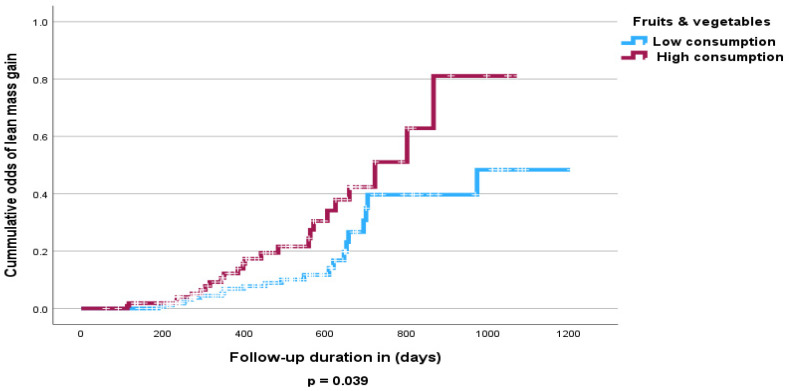
Kaplan–Meier curve of the effect of fruit and vegetable consumption on FFMM gain (high consumption >3.6 servings/day vs. low consumption <3.6 servings/day).

**Figure 5:**
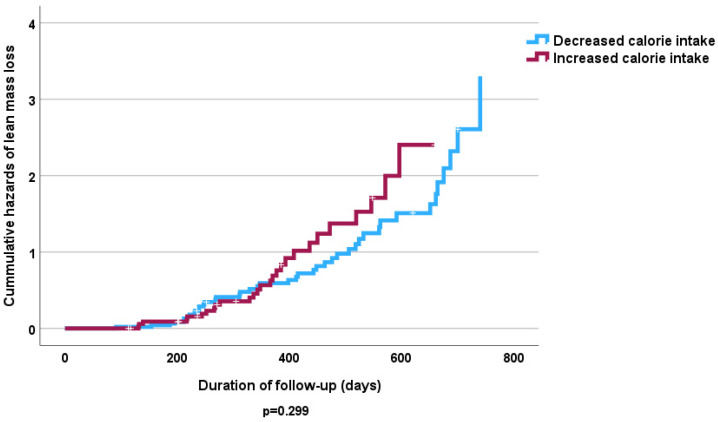
Kaplan–Meier curve of effect of dietary calorie intake at follow-up on FFMM loss.

**Figure 6:**
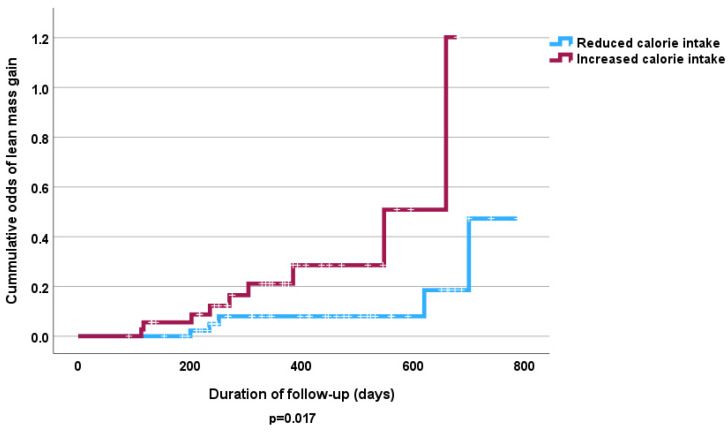
Kaplan–Meier curve of effect of dietary calorie intake at follow up on FFMM gain.

The Cox proportional hazards analysis revealed no independent significant influence of fruit and vegetable intake on lost FFMM compared to those that gained FFMM at follow-up (Table 5 and Table 6). However, the Kaplan–Meier curve shows that high consumption of fruits and vegetables was associated with increased odds of FFMM gain (*p* < 0.05) (Figure 3 and Figure 4). Similarly increased calorie intake showed a significant association with odds of increased FFMM (Figure 5 and Figure 6).

### 3.4. Associations Between Physical Activity and FFMM Gain or Loss at Follow-Up

**Figure 7:**
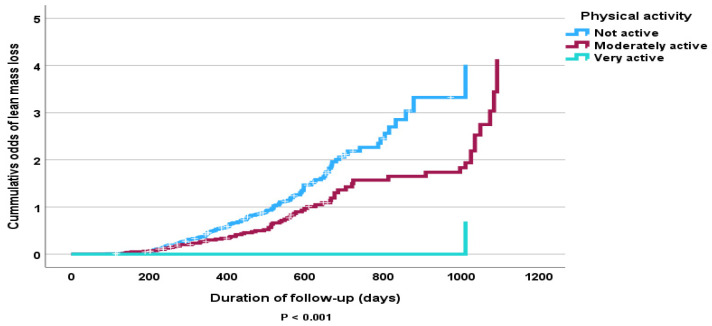
Kaplan–Meier curve of cumulative odds of FFMM loss at follow-up in all subjects according to levels of physical activity.

**Figure 8:**
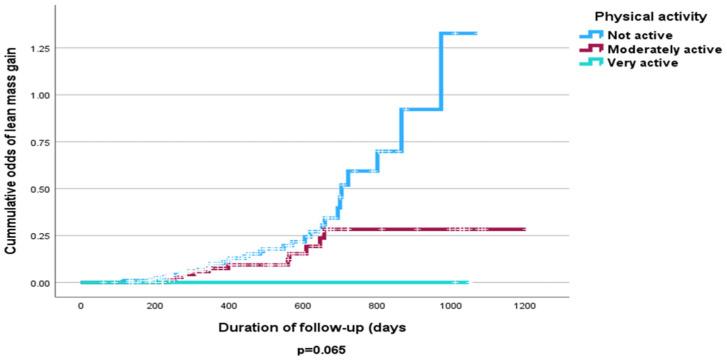
Kaplan–Meier of cumulative odds of FFMM gains at follow-up according to levels of physical activity.

The Cox proportional hazard model analysis revealed that increased physical activity was associated with significant FFMM gains and losses after adjusting for other prognostic indicators ([hazard ratio (95% CI): 0.49 (0.25, 0.97); *p* = 0.039; 0.55 (0.41, 0.73); *p* < 0.001, respectively] (Table 5 and Table 6). Only sex had a significant association with FFMM loss at follow-up (*p* = 0.003) (Table 5). Physical inactivity was particularly associated with FFMM loss (Figure 7). Figure 8 shows physical inactivity was associated with FFMM gain but the results are not statistically significant (*p* > 0.05).

## 4. Discussion

Our results show significant correlations between baseline fat–muscle mass ratio (FMR) and inflammatory and oxidative damage markers. We found no statistically significant differences in dietary protein and amino acid intake in subjects who gained FFMM compared to those who lost FFMM at baseline and follow-up. By contrast, physical activity, calories, and fruit and vegetable consumption were significantly associated with FFMM at baseline. In addition, increased physical activity was independently associated with significant FFMM gains. Physical inactivity was particularly associated with FFMM loss.

### 4.1. Fat and Muscle Mass and Obesity-Related Pathologies

Both fat mass and fat-free muscle mass play an important role in the development of cardiometabolic complications in obese individuals [6,7,8]. For example, some studies have revealed that increased muscle mass protects against obesity-related complications [18]. By contrast, increased fat mass increases the risk of hypertension, dyslipidemia, and impaired glucose regulation in obese subjects [6,9]. Furthermore, a significant diet-induced weight loss accompanied by a decrease in muscle mass and an increase in fat mass would increase the risk of sarcopenic obesity with associated low muscle mass and impaired muscle function [9,19,20,21].

Our results indicate an association between fat–muscle mass ratio (FMR) and metabolic risk factors. Increased fruit and vegetable consumption was also significantly associated with FFMM gains. These findings are intriguing because some of the possible mechanisms that relate obesity to increased complications including diabetes, hypertension, and CVS disease include oxidative damage and inflammation. Markers of inflammation correlate with markers of oxidative stress in obese individuals, which may be the reason for obesity-related complications. Recently, we reported that both elevated BMI and increased waist circumference are associated with increased metabolic risk factors in obese women; however, waist circumference is a stronger predictor than BMI [22]. Increased visceral fat has been particularly implicated in obesity-related complications [3]. In addition, our previous research findings suggested an association between increased fruit and vegetable consumption and a decrease in visceral fat. Other researchers have also reported that higher consumption of some fruits and vegetables is associated with lower abdominal fat gain [23]. Furthermore, beneficial effects of higher cereal fiber and fruit and vegetable intake on abdominal obesity prevention have been reported [24]. It is plausible that decreased inflammation and oxidative damage due to increased fruit and vegetable consumption favor lipolysis and lipid oxidation instead of fat storage in individuals with visceral obesity [3,25,26]. The association between visceral fat and FFMM as a result of intentional weight loss in obese subjects is clearly an area for further research.

### 4.2. Effects of Diet-Induced Weight Loss on Muscle Mass

Both fat and FFMM decrease due to weight loss achieved through a low-calorie diet. These results are different, however, for normal-weight people than for overweight and obese people [9]. There is some evidence that FFMM loss is higher among normal-weight people than among their overweight and obese counterparts. Furthermore, weight regain in normal persons promotes relatively more fat regain [27,28,29]. While overweight and obese individuals tend to lose less FFMM as a result of diet-induced weight loss compared with normal-weight individuals. The biological mechanisms responsible for diet-induced weight loss and decreases in FFMM are not well understood. This could be mediated through suppressed muscle protein synthesis or increased muscle breakdown. There is also some evidence that post-prandial protein synthesis is reduced by calorie restriction [30,31,32].

Although we found no statistically significant difference in dietary protein and amino acid intake in subjects who gained FFMM compared to those who lost FFMM, our results should be interpreted with caution because dietary intake in this study was assessed in a small sample both at baseline and follow-up. Nevertheless, current evidence suggests that high dietary protein intake helps preserve FFMM but may not improve muscle strength. It could also adversely affect metabolic function. This is clearly another area for further research.

### 4.3. Effects of Physical Activity/Inactivity on FFMM During Diet-Induced Weight Loss in Obese Individuals

Our study results also suggest that increased physical activity is independently associated with significant FFMM gain and that physical inactivity increases the odds of FFMM loss. This finding is not surprising given the wealth of evidence that progressive physical activity, when combined with a low-calorie diet, attenuates the weight loss associated with FFMM. However, the effect may be different for endurance type exercises [33,34,35,36,37] compared to progressive resistance exercises [38,39,40].

Different studies reached different conclusions regarding weight loss-induced FFMM changes when using a low-calorie diet and increased physical activity in combination or alone. Several possible explanations for these differences include small sample size, type of exercise, and the dose–response relationship between interventions and interindividual variability. Two important areas of the relationship and effect of physical activity on the etiology and treatment of obesity deserve special mention. There is evidence of an association between insulin resistance and physical inactivity, with some estimates of up to 10% of type 2 diabetes attributed to physical inactivity [41]. The biological mechanisms through which physical inactivity negatively impacts human health are not well understood [17]. In addition, recent evidence has revealed that both men and women overestimate their physical activity levels by around 44% and 138%, respectively [42].

Secondly, sedentary behavior is defined as long periods of time spent sitting or lying, which has independent adverse health effects [43]. The adverse health impact of physical inactivity and a sedentary lifestyle on women is different from that of men, possibly due to hormonal differences. Furthermore, a recent study on the benefit of physical activity in postmenopausal women revealed that postmenopausal women obtain less benefit from exercise. However, when supplemented with estrogen, that benefit was restored [44].

### 4.4. What Tasks Remain

More research is needed not only on different dietary strategies and physical activity type, intensity, and frequency in obesity treatments for muscle mass, but also on muscle strength and physical function given the study population’s age, gender, and ethnic background. Meanwhile, obese individuals should be encouraged to reduce sedentary behavior and physical inactivity. Weight loss and long-term weight maintenance will improve if sedentary behavior and physical inactivity are reduced and physical activity levels are increased. The core actions proposed by the WHO to prevent and treat obesity are a good start. These measures include (1) reducing commercial pressure on people particularly children to consume high energy products; (2) reducing sugar, salt and fat in manufactured products; (3) providing healthy food choices and means for increased physical activity in schools and the workplace; (4) better urban design and transport policies to promote and encourage cycling and walking [45].

### 4.5. Strengths and Limitations of the Study

The main limitation of our study was not using physical activity logs or pedometers, which are thought to be more accurate measures of physical activity than the validated physical activity interview questionnaire we used in this study. This limitation may have introduced bias given the reported evidence that people overestimate their physical activity levels [42]. Our total sample size is large and from a population with a high prevalence of obesity. However, dietary intake, namely protein and calorie intake, was measured in smaller subgroups than the total sample size of the studied cohort. When measuring FFMM, magnetic resonance and dual x-ray absorptiometry are more precise measures of fat and FFMM. Nevertheless, the Tanita bioelectrical impedance method is regarded as acceptable, feasible, and cost-effective for estimating body fat and mass [12]. Body composition measurements including FFMM were performed digitally and printed on a sheet with little room for observer error. We adjusted for important lifestyle factors in the analysis. Analysis of biological markers was carried out by a laboratory technician not involved in the recruitment or data collection of the study population.

## 5. Conclusions

In conclusion, this study demonstrated significant associations between increased physical activity and high dietary intake of calories, fruits and vegetables with FFMM preservation or gains. Although current public health advice is to increase physical activity and availability of healthy food choices to mitigate obesity related adverse health effects of FFMM loss, more research is needed to improve our knowledge in the following areas:

1. High protein vs. low calorie intake on muscle mass size and function;
2. Effect of high fruit and vegetable intake on lipid oxidation instead of fat storage in relation to FFMM change;
3. Impact of sedentary behavior/physical inactivity on fat and FFMM changes during intentional weight loss in obese individuals.

## Figures and Tables

**Table 1:** Baseline characteristics and anthropometric measurements of overweight compared to obese individuals, mean (SD) unless stated otherwise.

	Overweight [BMI = 25.1–29.9] (*n* = 284)	Obese [BMI ≥ 30] (*n* = 584)	*p*-Value
Age (years)	39 (12)	40 (12)	0.26
Females, n (%)	161 (56)	261 (44)	0.00
Waist circumference (cm)	89 (9.0)	103 (13)	0.00
Hip circumference (cm)	104 (11)	118 (13)	0.00
Fat mass (kg)	36 (7.0)	39 (7.0)	0.00
FFMM (kg) *	44 (6.0)	50 (8.0)	0.00
Fat-to-muscle mass ratio (%)	0.66 (0.21)	0.82 (0.17)	0.00
Lost FFMM at follow-up (kg)	40 (4) [n = 88]	46 (6) [n = 167]	0.00
Gained FFMM at follow-up (kg)	50 (6) [n = 39]	46 (6) [n = 49]	0.04

* Fat-free muscle mass (FFMM).

**Table 2:** Baseline anthropometric measurements of overweight and obese study populations stratified by sex, mean (SD).

	Overweight		Obese	
	Male	Female	Male	Female
Age (years)	45 (13)	37 (10) *	45 (1)	39 (11) *
BMI	28.2 (2)	28.4 (2)	28.2 (2)	34.6 (6) *
Waist circumference (cm)	84 (3)	85 (3)	104 (14)	103 (11)
Hip circumference (cm)	20 (38)	62 (51) *	83 (53)	101 (48) *
Fat mass (kg)	21 (7)	30 (8) *	31 (8)	40 (8) *
FFMM (kg)	57 (12)	45 (5) *	61 (14)	48 (6) *

* *p*-value < 0.05 for difference between male and females.

**Table 3:** Partial correlation between baseline fat–muscle mass ratio (FMR) and metabolic risk factors after adjusting for sex.

	Correlation Coefficient	*p*-Value
Systolic Blood pressure (mmHg)	r = −0.23	0.57
Diastolic blood pressure (mmHg)	r = −0.03	0.53
HBA1c (%)	r = −0.015	0.17
Glucose (mmol/L)	r = −0.30	0.02
hs CRP (mg/L) *	r = 0.23	0.00
TNFα (pg/mL)	r = 0.56	0.00
Antioxidant glutathione [GSH] (nmol/mL)	r = −0.03	0.66
Thiobarbituric Acid Reactive Substances (TBARS) (nmol/mL)	r = 0.56	0.00
Protein carbonyl (nmol/mg)	r = −0.14	0.00
Total Cholesterol (mmol/L)	r = −0.13	0.02
High density lipoprotein (mmol/L)	r = −0.09	0.11
Triglycerides (mmol/L)	r = −0.16	0.00

* C-reactive protein (hs-CRP).

**Table 4:** Logistic regression analysis of the relationship between lifestyle factors and other prognostic variables and FFMM at baseline.

Model	Unstandardized Coefficients	Standardized Coefficients	Sig.	95.0% Confidence Interval for B	
	B	Beta		Lower Bound	Upper Bound
Age (years)	1.09	0.13	0.00	0.00	0.00
Sex (male/female)	−2.90	−0.14	0.002	0.00	0.00
Occupation (employed, unemployed)	−1.53	−0.21	<0.001	0.00	0.00
Physical activity (very active, moderately active, not active)	1.22	0.09	0.02	0.18	2.26
fruits & vegetables consumption (servings/day)	3.87	0.21	0.00	2.26	5.47

**Table 5:** The Cox’s proportional hazard analysis of the relationship between lifestyle and other prognostic factors and FFMM gain at follow-up.

	Sig.	Exp(B)	95.0% CI for Exp(B)	
			Lower	Upper
Age (years)	0.88	1.00	1.00	1.00
Sex (male/female)	0.62	1.00	1.00	1.00
Occupation (employed, unemployed)	0.45	1.00	1.00	1.00
Physical activity (very active, moderately active, not active)	0.04	0.49	0.25	0.97
Number of hours doing house work/day	0.37	0.95	0.84	1.07
Fruits and vegetables consumption (servings/day)	0.05	1.82	1.00	3.33

**Table 6:** The Cox’s proportional hazard analysis of the relationship between lifestyle and other prognostic factors and FFMM loss at follow-up.

	Sig.	Exp(B)	95.0% CI for Exp(B)	
			Lower	Upper
Age (years)	0.42	1.00	1.00	1.00
Sex (male/female)	0.00	1.00	1.00	1.00
Occupation (employed, unemployed)	0.81	1.00	1.00	1.00
Physical activity (very active, moderately active, not active)	0.00	0.55	0.41	0.73
Number of hours doing house work/day	0.57	0.99	0.94	1.04
Fruits and vegetables consumption (servings/day)	0.81	1.04	0.78	1.37

## Data Availability

Data is available upon request to the corresponding author.
